# Editorial: Rise to the challenges in plastome phylogenomics

**DOI:** 10.3389/fpls.2023.1200302

**Published:** 2023-06-01

**Authors:** Wenpan Dong, Lianming Gao, Chao Xu, Yu Song, Peter Poczai

**Affiliations:** ^1^ School of Ecology and Nature Conservation, Beijing Forestry University, Beijing, China; ^2^ CAS Key Laboratory for Plant Diversity and Biogeography of East Asia, Kunming Institute of Botany, Chinese Academy of Sciences, Kunming, Yunnan, China; ^3^ State Key Laboratory of Systematic and Evolutionary Botany, Institute of Botany, Chinese Academy of Sciences, Beijing, China; ^4^ Key Laboratory of Ecology of Rare and Endangered Species and Environmental Protection (Ministry of Education), Guangxi Normal University, Guilin, Guangxi, China; ^5^ Guangxi Key Laboratory of Landscape Resources Conservation and Sustainable Utilization in Lijiang River Basin, Guangxi Normal University, Guilin, Guangxi, China; ^6^ Botany Unit, Finnish Museum of Natural History, University of Helsinki, Helsinki, Finland; ^7^ Museomics Research Group, Helsinki Institute of Life Science (HiLIFE), Helsinki, Finland

**Keywords:** plastome phylogenomics, incomplete lineage sorting, introgression, species tree, phylogenetic discordance

The plastome has a quadripartite organization, encodes about 114 unique genes, and has earned its reputation in plant phylogenomics for ease of obtaining and handling plastome data and its considerable phylogenetic information content (Mehmood et al., 2020). The power of plastome phylogenomics has been exemplified by reconstructing deep to shallow phylogenetic relationships, deducing reticulate evolutionary histories, and phylogenetically placing taxa (Watson et al., 2020; Guo et al., 2023). Despite the upsurge in popularity, decades of studies have raised controversy on the advantages and shortcomings of plastome phylogenomics. Features such as the inheritance characteristics of plastomes, recombination, gene transfer, or specific evolutionary patterns may result in limited or, even worse, incorrect inferences (Gonçalves et al., 2019). Whereas tree discordances are often the primary indication for such problems, the underlying mechanisms should be more extensively explored (Gonçalves et al., 2020; Rose et al., 2021; Doyle, 2022; Kao et al., 2022).

In this Research Topic, we collected studies conducted on solid and thorough plastome phylogenomics throughout the plant tree of life. Chen et al. studied *Isodon* (Schrad. ex Benth.) Spach, a large genus of the Lamiaceae family important for its medicinal properties. Rapid radiation has made it difficult to distinguish species, especially within Clade IV, which contains over 80% of taxa. To elucidate the phylogenetic relationships within the genus, their study used plastome and nrDNA sequences to reconstruct the phylogeny of approximately 80% of the species. While the results revealed major lineages consistent with previous studies, incongruences were found due to insufficient phylogenetic signal, hybridization, and plastome capture. They revealed that more data from the nuclear genome are needed to resolve relationships within Clade IV and highlighted that nutlet morphology can distinguish the four major clades of *Isodon*.

Xi et al. utilized 19 newly generated plastomes of *Carya* Nutt. (Juglandaceae) species, including the critically endangered species *C. poilanei*, to explore maternal relationships among the subclades of the genus to more comprehensively evaluate plastid genomes, for which variation has not been thoroughly characterized. The results indicated remarkable differences in several plastome features to be highly consistent with the EA-NA disjunction, highlighting the importance of full-length plastomes as an ideal tool for exploring maternal relationships among *Carya* subclades and potentially in other outcrossing perennial woody plants to resolve inter-specifc phylogenetic relationships.

Plastome data were used by Wang et al. to estimate genetic diversity and divergence times, rebuild biogeographic history, and predict potential distribution of the genus *Coptis* Salisb. (Ranunculaceae). With 15 recognized species and high medicinal value, *Coptis* has a conspicuous taxonomy with a unique evolutionary position, distribution pattern, and conservation significance. They revealed that the low nucleotide diversity of *Coptis* plastomes is 0.0067 and the hotspots are located in the *ycf*1 gene. *Coptis* originated in North America and the Japanese archipelago and has a typical Eastern Asian and North American disjunct distribution pattern. The most suitable climatic conditions for *Coptis* were identified, and the study provided insights for future conservation efforts.

Han et al. investigated the genetic structure of *Pistacia chinensis* Bunge (Anacardiaceae), an important tree crop in China known for its high fruit oil content. Through analysis of the plastome and nuclear SNPs of 39 individuals across China, their study identified five clades of *P. chinensis* and the occurrence of hybridization events between highly divergent samples in the subclades. The study suggested that there is much unlocked genetic diversity in this recently domesticated species, which could be exploited for Chinese pistache improvement.

Liu et al. highlighted the complexity of the taxonomic relationships among *Atractylodes* DC (Asteraceae) species, which are cultivated as medicinal herbs in China, Japan, and Korea. The study used high-throughput sequencing to obtain concatenated nuclear ribosomal DNA sequences and plastid genomes from 24 plant samples from five species of *Atractylodes* located in China, of which 23 belonged to members of the *A. lancea* complex. The study identified a mixed clade among this species complex, suggesting the possibility of hybridization or gene introgression.

Zeng et al. analyzed the chloroplast genomes of 123 varieties of mulberry plants from six different species. The study revealed that the analyzed *Morus* taxa should be classified into six species, with two subspecies for *M. alba*. Their research offered valuable insights into the classification, domestication, and breeding improvement of mulberry.

Xiao et al. successfully reconstructed the phylogenetic backbone of *Clematis* L., one of the largest genera of Ranunculaceae, using transcriptome data and plastid genome sequences, nuclear SNP datasets, and single-copy nuclear orthologous genes assembled from genome skimming data. The study found that the assembled datasets could effectively resolve the phylogeny of *Clematis*, showing that rapid species radiation may have caused incomplete lineage sorting, while frequent interspecific hybridization events may have led to cyto-nuclear discordances. The research also provides insights into genome partitioning strategies for future phylogenomic studies of other plant taxa.

Wu et al. focused on the phylogenetic relationships of the family Convolvulaceae, which includes morning glories, bindweeds, and plants of economic importance, such as traditional medicines, ornamentals, and vegetables. They confirmed the monophyly of the family and provided insights into its phylogenomic relationships. The positions of some genera, including *Cuscuta* L. and *Erycibe* Roxb., were uncertain due to variable nucleotide substitution rates. The study also detected numerous plastomic rearrangements, including inversions, duplications, losses of genes, and introns. A rare example of gene transfer from mitochondria to plastids in angiosperms was found in the *Jacquemontia* Choisy plastome.

The taxonomy and phylogenetic relationships of the subgenus *Cerasus*, which includes over 100 species of the genus *Prunus* L. (Rosaceae), are unclear. To address this, Wan et al. reconstructed the phylogenetic tree for 11 species from *P.* subg. *Cerasus* using plastid genome sequences. Phylogenetic analysis revealed the true cherry group to be most similar to the flora of China, with *P. mahaleb* L. forming a distinct subclade. The study provided new insights and potential molecular markers for further research.

The comprehensive evaluation of plastome sequences among plant lineages revealed tree discordance with nuclear genes, elucidating biological and computational factors affecting tree topologies. For phylogenetic studies, a more balanced understanding of the evolutionary history of plastid genes is crucial. The phylogenetic signal of plastid sites and genes varies in support of alternate topologies at various taxonomic levels, as research published in the Research Topic have demonstrated. The disagreement between topologies supported by the greatest number of genes or sites and the topologies supported by the strongest phylogenetic signal may be an indicator of how the phylogenetic signal’s variability affects various phylogenetic inference techniques. Complete plastid phylogenies still need to be explored below the order and family levels, despite extensive exploration of this genomic compartment and thorough knowledge of numerous plant phylogenetic nodes. In addition to the common use of plastome data to reconstruct evolutionary connections of different plant groups, we propose comparing plastid phylogenies with nuclear data to uncover possible differences within genomic compartments.

## Author contributions

All authors listed have made a substantial, direct, and intellectual contribution to the work. All authors of the manuscript have read and agreed to its content and are accountable for all aspect of the accuracy and integrity of the manuscript in accordance with ICMJE criteria.
